# Accuracy of Magnetic Resonance Imaging in Staging Rectal Cancer with Multidisciplinary Team: A Single-Center Experience

**DOI:** 10.7150/jca.32685

**Published:** 2019-10-21

**Authors:** Linzhen Yu, Liuhong Wang, Yinuo Tan, Hanguang Hu, Li Shen, Shu Zheng, Kefeng Ding, Suzhan Zhang, Ying Yuan

**Affiliations:** 1 Department of Medical Oncology, the Second Affiliated Hospital, Zhejiang University School of Medicine, Hangzhou 310009, China.; 2 Department of Radiology, the Second Affiliated Hospital, Zhejiang University School of Medicine, Hangzhou 310009, China.; 3 Department of Radiation Oncology, the Second Affiliated Hospital, Zhejiang University School of Medicine, Hangzhou 310009, China.; 4 Department of Colorectal Surgery, the Second Affiliated Hospital, Zhejiang University School of Medicine, Hangzhou 310009, China.; 5 Cancer Institute (Key Laboratory of Cancer Prevention and Intervention, China National Ministry of Education), the Second Affiliated Hospital, Zhejiang University School of Medicine, Hangzhou 310009, China.

**Keywords:** Rectal cancer, Magnetic resonance imaging, Multidisciplinary team, Preoperative stage

## Abstract

**Purpose**: To investigate the accuracy of magnetic resonance imaging (MRI) in preoperative staging diagnosis for rectal cancer with multidisciplinary team (MDT) discussion.

**Methods**: The retrospective study included 377 patients of rectal cancer with preoperative MRI staging from February 2015 to April 2018, in which 137 patients (36 received MDT discussion) received neoadjuvant therapy, 240 did not (97 received MDT discussion) and direct surgery was given. With postoperative pathological stage as the standard, the accuracy of MRI in preoperative staging for rectal cancer with MDT discussion was compared with non-MDT.

**Results**: For direct surgery group, 21 out 97 (21.6%) patients changed their therapy strategy due to the change of the stage assessment after MDT. The accuracy of MRI for the diagnosis of preoperative N stage with MDT was significantly higher than those without MDT (56.2% vs. 42.1%, P=0.021). And for those without lymph node metastasis, the accuracy of MRI was higher after MDT (61.2% vs. 37.8%, P=0.009). For neoadjuvant therapy group, 7 out of 36 (19.4%) patients altered their therapy after MDT because of the changed stage. MDT improved the accuracy of restaging N stage with MRI (70.0% vs. 33.3%, P=0.003). The accuracy of MRI in staging T stage seemed not improved after MDT in both groups.

**Conclusions**: In conclusion, MDT discussion increased the accuracy of MRI in preoperative staging diagnosis for rectal cancer. This mode could give a more accurate clinical stage of patients, which was in favor of choosing a preferable therapy strategy.

## Introduction

Colorectal cancer is one of the most common cancers worldwide 1. In 2014, the incidence rate and mortality rate of colorectal cancer were 27.8/100000 and 13.3/100000 respectively in China 2. In colorectal cancer, 30% is rectal cancer which is within 15 centimeters of the edge of the anus 3. The treatment of rectal cancer depends on the stage, and whether patients need to receive neoadjuvant therapy is based on the depth of infiltration and the lymph node metastasis 4. Magnetic resonance imaging (MRI) plays an important role in evaluating the stage of rectal cancer. MRI shows precise anatomy of the rectum and mesenteric fascia, and predicts circumferential resection margin and tumor stage accurately. It also predicts the risk of local recurrence and simultaneous metastasis or heterochronous metastasis. Doctors can make a better therapy strategy according to the patients' preoperative assessment by MRI 5-7. During or after the neoadjuvant therapy, restaging is usually performed to evaluate the therapeutic effect and adjust the treatment 4. MRI in diagnosing the stage of rectal cancer is recommended by the European Society of Gastrointestinal and Abdominal Radiology (ESGAR) 8.

Usually, the multidisciplinary team (MDT) of colorectal cancer consists of several departments; imaging and pathology make the diagnosis while colorectal surgery, oncology, radiotherapy and palliative care provide the therapy 9, 10. The MDT mode can improve the survival of rectal cancer, and is recommended by many guidelines 11-13. However, there is few researches report the importance of MDT in the assessment of MRI and the influence of MDT for accurate staging and therapy strategy selecting. So we did a retrospective analysis of patients in our hospital to investigate the accuracy of MRI in preoperative staging diagnosis for rectal cancer with MDT.

## Methods

Inclusion criteria of participants were: (1) Patients received radical resection of rectal cancer and were histologically confirmed as rectal cancer by postoperative pathology. (2) The results of the preoperative staging diagnosis by MRI were recorded. (3) Patients with distant metastasis were excluded according to the results of their abdominal enhanced computerized tomography (CT), chest high resolution CT and even positron emission tomography computerized tomography (PET-CT).

According to the criteria, 377 rectal cancer patients with preoperative MRI diagnosis were admitted from February 2015 to April 2018 in the Second Affiliated Hospital of Zhejiang University School of Medicine. Patients were divided into direct surgery group and neoadjuvant therapy group. In direct surgery group, patients received surgery directly after diagnosis, while patients in the neoadjuvant therapy group received neoadjuvant therapy. 97 out of 240 patients in direct surgery group received MDT discussion. 137 patients were in neoadjuvant therapy group, in which 36 received MDT discussion. This study was approved by Ethics Committee of the Second Affiliated Hospital of Zhejiang University School of Medicine.

The final assessment criteria were the postoperative pathological results, which were based on the 8th edition of the AJCC cancer staging manual 14. In both group, we compared the most recent preoperative stage diagnosed by MRI with the pathological stage.

In patients with MDT, the MRI for staging and restaging were assessed by the radiologists in the MDT team of rectal cancer, together with colorectal surgeon, radiology oncologist and medical oncologist. And in patients without MDT, radiologists who were not part of MDT team made the diagnosis of MRI for staging and restaging alone. All the radiologists were at least senior attending doctors who reached the average professional level. The criteria of MRI for assessing T stage were based on the differences of signal intensity between submucosa, muscular layer, mesorectum and tumor in T2 phase. And the criteria of lymph node involvement were short diameter of node more than 8mm, blurred borders, irregular morphology and uneven signal or echo 15, 16.

SPSS20.0 statistical software was used for analysis. According to the postoperative pathological results, we calculated the sensitivity, specificity, negative predictive value, positive predictive value and the accuracy of MRI. The formulas were shown as below:

Sensitivity = (true positives)∕(true positives + false negatives);

Specificity = (true negatives)∕(true negatives + false positives);

Negative predictive value = (true positives)∕(true positives + false positives);

Positive predictive value = (true negatives)∕(true negatives + false negatives).

Comparison of qualitative variables was performed by *χ*^2^ test, and P < 0.05 was considered statistically significant.

## Results

### The accuracy of MRI in direct surgery group

With MDT, the accuracy of MRI for diagnosing preoperative T stage was higher than those without MDT with marginal statistical significance (84.2% vs. 76.0%, P=0.077, Table 1). For pathological T0 to T2 stage, the accuracy of MRI was 56.0% after MDT, and was 36.5% without MDT discussion (P=0.086). For pathological T3 to T4 stage, there was no significant difference of the accuracy for MRI between MDT and non-MDT mode. As for preoperative N stage, the accuracy of MRI for diagnosing preoperative N stage was significantly higher than without MDT (56.2% vs. 42.1%, P=0.021). And for those without lymph node metastasis, the accuracy of MRI was improved after MDT (61.2% vs. 37.8%, P=0.009). The accuracy of MRI after MDT was not different from non-MDT in patients with lymph node metastasis.

**Figure 1:**
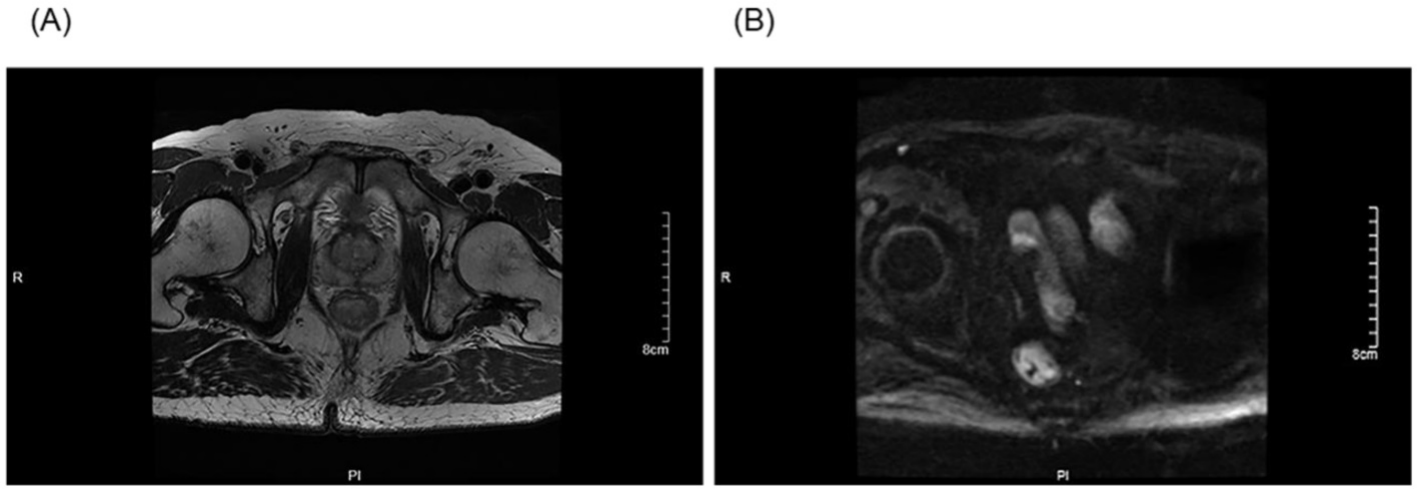
The preoperative stage was corrected after an MDT discussion: The T stage of a patient was corrected from T3 to T2 after an MDT discussion (A). The N stage of a patient was corrected from N2 to N0 after an MDT discussion (B).

And 21 out 97 (21.6%) patients changed their therapy strategy due to the change of the stage assessment after MDT (Figure 1). Sensitivity, specificity, negative predictive value and positive predictive value of MRI were shown in Table 2.

### The accuracy of MRI in neoadjuvant therapy group

The accuracy of MRI in diagnosing stage of rectal cancer with or without MDT was shown in Table 3. For assessing T stage after neoadjuvant therapy, the accuracy of MRI with MDT discussion was not significantly different from non-MDT (70.0% vs. 78.6%, P=0.293). And the accuracy of N stage was higher with MDT than without (70.0% vs. 33.3%, P=0.003). For patients at N0, compared with non-MDT, MDT improved the accuracy of MRI for restaging (72.2% vs. 31.6%, P=0.003). 7 out of 36 (19.4%) patients altered their therapy after MDT because of the changed stage. Sensitivity, specificity, negative predictive value and positive predictive value were shown in Table 4.

## Discussion

Our study investigated the role of MDT discussion in diagnosing preoperative staging of rectal cancer with MRI. The results suggested that MDT improved the accuracy of MRI, and about 20% patients changed their therapy due to the corrected clinical stage.

Rectal cancer is a common malignant tumor, which often occurs in the elderly. The mortality rate of rectal cancer is 4-10/10000 per year, which is one of the major causes of cancer-related death 7, 17. The prognosis of rectal cancer is related to age, general condition of the patient, depth of tumor invasion, lymph node metastasis, circumferential resection margin and invasion of extravascular vascular 18-20. The 5-year survival rate of rectal cancer is 66.6%, and localized cancer 88.2%, regional metastasis 70.0%, distant metastasis 14.0% 21. The clinical stage of rectal cancer is one of the factors that determine the patients whether to receive surgery directly, or neoadjuvant therapy followed by radical resection, or palliative chemotherapy, or radiotherapy. And the response evaluation of neoadjuvant therapy may change the following treatment 22. Therefore, preoperative evaluation of rectal cancer is important for the choice of treatment and prediction of prognosis.

The gold standard for diagnosing rectal cancer is endoscopy with biopsy for histopathological confirmation. And imaging examinations play an important role in the diagnosis of rectal cancer. Imaging examinations for rectal cancer include CT, MRI, endorectal ultrasonography (ERUS), and PET-CT 7, 23. The strength of MRI is the ability to identify the mesorectal fascia, which makes it possible to preoperatively accurately identify those complete surgical excision are infeasible 24. MRI can identify mucosa and muscle with different signal characteristics, and assess T stage based on signal intensity in and out the submucosa of the rectal wall. Lymphatic involvement assessment is based on the signal in mixed nodules, boundary irregularity, and nodule size. The effect of neoadjuvant therapy is assessed based on the proportion of residual tumor cells in the fibrotic matrix 4, 22. A meta-analysis showed that the sensitive of MRI for diagnosing T and N stage of rectal cancer were 75% and 71% respectively 15. Brown et al revealed that compared with pathological results, the coincidence rate of MRI in diagnosing T stage was 94%, and N stage was 85%. MRI was of poor assessment in lymph nodes relatively 25. Our study suggested the similar results. In addition, the accuracy of MRI in restaging after neoadjuvant therapy was relatively low. The reason was that the edema, inflammation, necrosis, and fibrosis of tissue made it indistinguishable from tumors after chemoradiotherapy 4.

The goal of MDT is to make personalized medical treatment according to the individual and tumor characteristics of patients. MDT discussion is able to alter therapy and improve the prognosis. A single-center study in Scotland showed that in patients with non-surgical non-small cell lung cancer, more patients received chemotherapy rather than palliative care after MDT 9. MacDermid revealed that the proportion of postoperative adjuvant chemotherapy in colorectal cancer patients was increasing significantly after MDT with the improved 3-year survival 12. And for metastatic colorectal cancer, the 3- and 5-year survival improved after MDT 26. Burton et al showed the positive rate of circumferential resection margin in rectal cancer reduced after MDT 10. MDT decreased the local recurrence rate of rectal cancer, which might because of the more precise diagnosis of preoperative stage in MDT mode 27. A population-based study suggested in MDT mode, rectal cancer patients received more preoperative MRI examination and the TNM stage was more complete 28. In our study, the accuracy of MRI in staging rectal cancer increased after MDT.

The major reason of the improvement might be that the subspecialties of radiologists in MDT are the imaging diagnosis and research of colorectal cancer. That makes it possible for radiologists in MDT to make more accurate stage assessment based on the background that they are more familiar with the patients' clinical information. Together, the participants of MDT made the more accurate diagnosis. There were some limitations in our study and might result in bias. Patients were mainly at stage T3, and the pathology department tended to diagnose T3 rather than T4a, which made it difficult to compare the accuracy of MRI between MDT and non-MDT in diagnosing patients at T3 to T4. And the number of T0 to T2 was small, which made it possible that data analysis was not significant. These factors might account for why the accuracy of T stage was not improved after MDT.

In conclusion, our single-center experience showed that the accuracy of MRI in staging rectal cancer was improved after MDT discussion. And 21.6% patients in direct surgery group, 19.4% in neoadjuvant therapy group changed their therapy strategy due to the change of the stage assessment after MDT. Doctors are able to choose the suitable treatment strategy for rectal cancer patients based on the accurate diagnosis after MDT discussion and achieve the goal of improving patients' outcome.

## Figures and Tables

**Table 1:** The accuracy of MRI in direct surgery group

	T stage	T0-2	T3-4	N stage	N-	N+
MDT	84.2%	56.0%	94.3%	56.2%	61.2%	50.0%
Non-MDT	76.0%	36.5%	93.3%	42.1%	37.8%	47.9%
P value	0.077	0.086	0.156	0.021	0.009	0.495

N(-): There is no regional lymph nodes metastasis; N(+): There is lymph nodes metastasis.

**Table 2:** The sensitivity, specificity, positive predictive value and negative predictive value of MRI in direct surgery group

	T stage		N stage	
	MDT	Non-MDT	MDT	Non-MDT
sensitivity	0.93	0.81	0.73	0.88
specificity	0.58	0.56	0.61	0.38
PPV	0.87	0.88	0.60	0.51
NPV	0.74	0.42	0.73	0.80

PPV: positive predictive value; NPV: negative predictive value.

**Table 3:** The accuracy of MRI in neoadjuvant therapy group

	T stage	T0	T1-4	N stage	N-	N+
MDT	70.0%	0%	93.3%	70.0%	72.2%	50.0%
Non-MDT	78.6%	5.9%	100%	33.3%	31.6%	38.1%
P value	0.293	0.773	0.187	0.003	0.003	0.640

N(-): There is no regional lymph nodes metastasis; N(+): There is lymph nodes metastasis.

**Table 4:** The sensitivity, specificity, positive predictive value and negative predictive value of MRI in neoadjuvant therapy group

	T stage		N stage	
	MDT	Non-MDT	MDT	Non-MDT
sensitivity	0.93	1.00	0.50	0.90
specificity	0.00	0.05	0.74	0.32
PPV	0.73	0.78	0.17	0.33
NPV	0.00	1	0.93	0.90

PPV: positive predictive value; NPV: negative predictive value.

## Abbreviations

MRI: magnetic resonance imaging
MDT: multidisciplinary team
ESGAR: European Society of Gastrointestinal and Abdominal Radiology
CT: computed tomography
ERUS: endorectal ultrasonography
PET: positron emission tomography
PPV: positive predictive value
NPV: negative predictive value
